# Tumor-Associated Granulomas Preceding a Diagnosis of Thoracic Sarcoidosis: A Retrospective, Single-Center Cohort Study

**DOI:** 10.3390/jcm10184151

**Published:** 2021-09-15

**Authors:** Patricia Muñoz-Hernández, Mariel Valdivia-Mazeyra, Julio Ancochea, Tamara Alonso Pérez, Olga Rajas, Claudia Valenzuela, Susana Hernández-Muñiz, Amparo Esteban-Peris, José A. Jiménez-Heffernan

**Affiliations:** 1 Department of Pathology, University Hospital La Princesa, Diego de León, 62, 28006 Madrid, Spain; patricia.munoz@salud.madrid.org (P.M.-H.); mariel.valdivia@salud.madrid.org (M.V.-M.); 2 Department of Pneumology, University Hospital La Princesa, Diego de León, 62, 28006 Madrid, Spain; julio.ancochea@uam.es (J.A.); talonsop@salud.madrid.org (T.A.P.); olga.rajas@salud.madrid.org (O.R.); claudia.valenzuela@salud.madrid.org (C.V.); 3 Department of Radiology, University Hospital La Princesa, Diego de León, 62, 28006 Madrid, Spain; susana.hernandezm@salud.madrid.org; 4 Department of Radiology, University Hospital La Moraleja, Avda. Francisco Pi y Margall, 81, 28050 Madrid, Spain; mdesteban@sanitas.es

**Keywords:** granulomas, neoantigens, neoplasia, sarcoidosis, sarcoid-like reaction, tumoral antigens

## Abstract

There is a relationship between systemic sarcoidosis (SS) and malignancy. Sarcoidosis results from an exaggerated immune response in genetically susceptible individuals. In oncologic patients with sarcoidosis, tumoral antigens and antineoplastic treatment are considered potential triggering factors. The observation of a patient with granulomas in a parotid carcinoma who later developed SS led us to review the previous tumors of patients with SS. The aim of the study is to see whether granulomas were already present in the tumors that preceded sarcoidosis. We identified 196 sarcoidosis patients, 47 of whom had previously had a tumor. We were able to review 29 cases, 12 of which showed tumor-associated granulomas (TAGs) (41.4%). This ratio is much higher than that of the normal population (4.4–13.8). We analyzed five control patients without sarcoidosis for each tumor. In conclusion, we observed an increased number of TAGs in patients who later developed SS. This finding reinforces a pathogenic relationship between SS and neoplasia. The histology of tumors in patients with SS should be reviewed in an attempt to identify granulomas.

## 1. Introduction

The diagnosis of systemic sarcoidosis (SS) is based on three major criteria: a compatible clinical presentation, the finding of non-necrotizing granulomatous inflammation, and the exclusion of alternative causes of granulomatous disease [1]. Obtaining tissue material for the detection of granulomas has been a limiting condition historically for the diagnosis of SS. However, the introduction of endobronchial ultrasound-guided transbronchial needle aspiration (EBUS-TBNA) has facilitated the access to mediastinal nodes enormously and has proved a reliable method for detecting non-necrotizing granulomas, thus simplifying the diagnosis [2,3]. Several studies have demonstrated a relationship between SS and malignant tumors [4,5,6,7,8]. Sarcoidosis can occur before, during, or after cancer diagnosis [4,6]. For instance, patients with known SS have a 2–3-fold risk of developing a malignant neoplasm, and the reasons for this increased risk are not clear [4]. While SS is a relatively well-known condition, a sarcoid-like reaction (SLR) is defined as a non-necrotizing granulomatous reaction occurring under conditions which do not meet the diagnostic criteria for SS [1,4]. Cases of sarcoidosis following malignancy are clinically similar to idiopathic sarcoidosis [7]. Regarding malignancy, patients with sarcoidosis must be distinguished from those in whom an SLR is histologically seen in the primary tumor or draining lymph nodes, as the latter do not fulfill the criteria for SS. 

Sarcoidosis is considered a disorder resulting from an exaggerated immune response to an unknown antigen occurring in genetically susceptible patients [9]. In oncologic patients who develop SS, tumoral antigens are considered the most likely triggering factor [4,5,6,7,8]. In addition, antineoplastic treatment can also play a causative role [4,5,6,7,8]. Although the possibility of an immune reaction against tumoral antigens is likely, we must consider that individuals who develop SS may simply be prone to a granuloma-forming response to a variety of antigens. Therefore, the subsequent development of thoracic sarcoidosis observed in these patients is not necessarily related to tumoral antigens. Routine imaging studies in the follow-up of oncologic patients result in the detection of sarcoidosis in the early stages [7]. As the possibility of metastatic disease cannot be ruled out, most of these patients undergo pathologic analysis of the mediastinal lymph nodes. 

The clinical observation of a patient with an extensive non-necrotizing granulomatous reaction in a parotid acinic cell carcinoma who 15 months later developed SS led us to study other patients with sarcoidosis and pre-existent tumors. The presence of an SLR in neoplasms or draining nodes is a well-known pathologic phenomenon but has not been directly related to the subsequent development of sarcoidosis [10,11]. In the normal population without SS, SLRs occur in 4.4% of carcinomas, in 13.8% of patients with Hodgkin’s disease, and in 7.3% of cases of non-Hodgkin lymphomas [10]. They are extremely rare in sarcomas [10]. 

## 2. Materials and Methods

Based on the above-mentioned criteria for SS [1], we performed a retrospective study at the University Hospital La Princesa of patients in whom a diagnosis of mediastinal or pulmonary sarcoidosis with pathologic evidence of granulomas was present. The study protocol was approved by the Ethics Committee of the University Hospital La Princesa, Madrid, Spain (code: 4238). Lung and mediastinal lymph nodes are among the major organs affected by sarcoidosis, so we reviewed the pathology files using a search tool for cross-queries with words such as “mediastinal”, “lymph node”, “pulmonary”, “granuloma”, “sarcoidosis”, or “sarcoid”. The search covered the period from January 2000 to December 2020. We then reviewed the cases in collaboration with the Pneumology Department and selected those patients that fulfilled a diagnosis of thoracic sarcoidosis. Of all the patients diagnosed with mediastinal or pulmonary sarcoidosis, we searched for those who had previously had a neoplasm. From the Pathology Department files, we retrieved the pathologic material. An important aspect of the study was to locate, in those selected patients with sarcoidosis and a previous tumor, chest-imaging studies at the moment of tumor resection to demonstrate no mediastinal or pulmonary abnormalities. This was performed to exclude cases of asymptomatic pre-existing sarcoidosis. All patients were adults as the University Hospital La Princesa does not treat pediatric patients. 

We reviewed all histologic and cytologic slides corresponding to the diagnosis of sarcoidosis and a previous tumor. Three pathologists examined the pathologic samples together and only considered those cases they unanimously agreed upon. We used a semiquantitative scale to evaluate the quantity of granulomas: rare, when only isolated granulomas were seen; moderate, when they were easy to identify; and abundant, when there was a remarkable finding. We also classified the distribution of granulomas as intratumoral, peritumoral, or both. For each tumor preceding a sarcoidosis diagnosis, we selected five controls from the same entity in patients without sarcoidosis. Meanwhile, as no other cases were available regarding sebaceous lymphadenoma, we used five Warthin tumors and five pleomorphic adenomas as controls. In these cases, the same three pathologists performed a detailed search for granulomas.

## 3. Results

**Figure 1:**
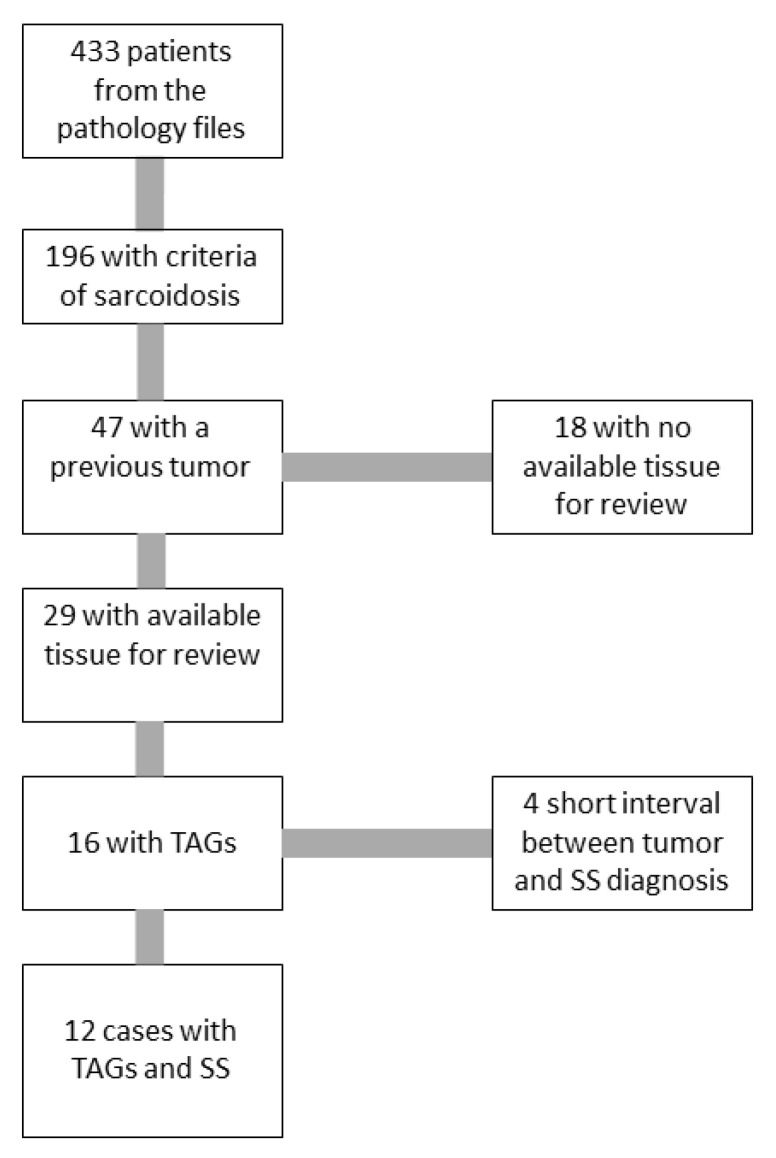
Flowchart showing the selected patients. The total of 433 was obtained from the pathology files after a search tool for cross-queries with words such as “mediastinal”, “lymph node”, “pulmonary”, “granuloma”, “sarcoidosis”, or “sarcoid”. TAGs—tumor-associated granulomas, SS—systemic sarcoidosis.

From the first search, 433 patients were identified, of whom 196 fulfilled the criteria for SS [1]. The medical records of 47 (23.9%) patients revealed the existence of a previous tumor. The pathologic files of our department contained the histologic material of 29 of these 47 patients. The tumors of the remaining 18 patients were treated in different medical centers and the histologic slides were unavailable for review. We reviewed the complete pathologic material of these 29 patients, including the mediastinal or pulmonary samples, where non-necrotizing granulomas were observed, as well as the previous tumor. We found TAGs in 16 of the 29 tumors. Four cases had to be excluded as the time interval between the diagnosis of SS and the previous tumor was too short (less than 8 weeks) and the possibility of simultaneous presentation could not be excluded. The remaining 12 constitute the basis of this study (Figure 1), with Table 1 summarizing their main clinicopathologic features. 

**Figure 2:**
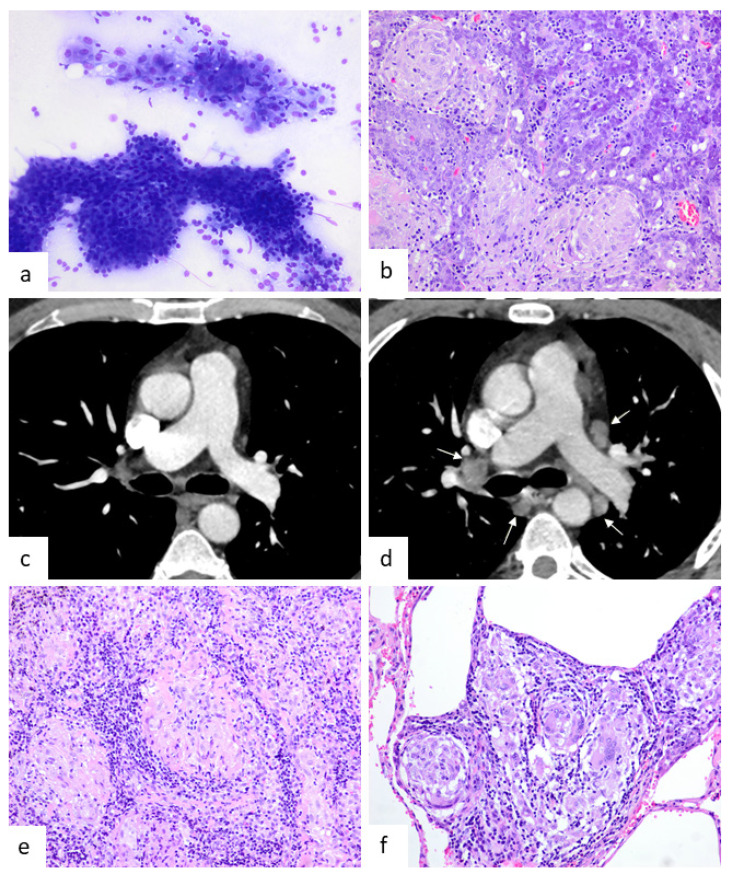
Images from patient 1 that motivated the study. (**a**,**b**) Parotid acinic cell carcinoma showing granulomas in a fine needle aspiration sample and subsequent surgical specimen (Diff-Quik, ×400, HE, ×400, respectively). (**c**,**d**) The first CT scan (**c**) was performed 6 months after surgery and shows no abnormalities. A second CT scan (**d**) performed 15 months after surgery shows evident mediastinal lymphadenopathies (gray arrows) that were absent in the previous one. (**e**,**f**) Mediastinal lymph node and lung biopsy showing numerous non-necrotizing granulomas (HE, ×400).

**Figure 3:**
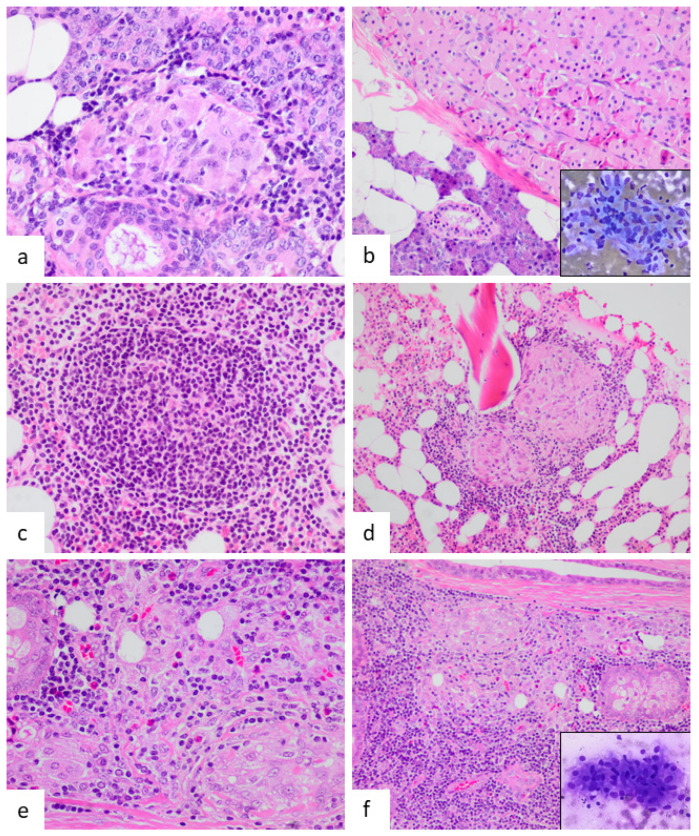
(**a**,**b**) Peritumoral granulomas in the case of parotid oncocytoma (case 2) (HE, ×600 and ×200, respectively). The inset reveals a granuloma in a fine needle aspiration sample of an enlarged mediastinal lymph node performed 30 months later (Diff-Quik, ×400). (**c**,**d**) Bone marrow showing follicular lymphoma involvement and numerous non-necrotizing granulomas (case 3) (HE, ×400 and ×200, respectively). (**e**,**f**) Two images of sebaceous lymphadenoma showing intratumoral granulomas (case 5) (HE, ×200 and ×400).

**Figure 4:**
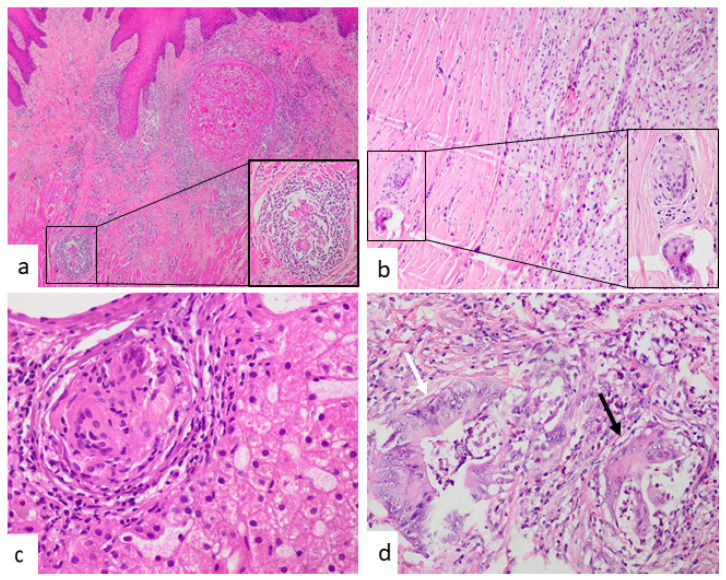
(**a**) Peritumoral granulomas in a squamous cell carcinoma of the tongue. Granulomas are peritumoral and have no relationship with keratin material (case 6) (HE, ×40). The inset highlights the absence of foreign body component and non-necrotizing morphology. (**b**) Peritumoral granuloma in a case of cutaneous neurofibroma (case 8) (HE, ×100, inset ×400). (**c**) Hepatic granuloma in a peritumoral location. The granuloma is surrounded by hepatocytes without visible neoplastic cells (case 7) (HE, ×600). (**d**) Intratumoral granuloma in a case of rectal adenocarcinoma. The granuloma (black arrow) is in close relationship with malignant glands present at the left (white arrow) (case 9) (HE, ×400).

**Figure 5:**
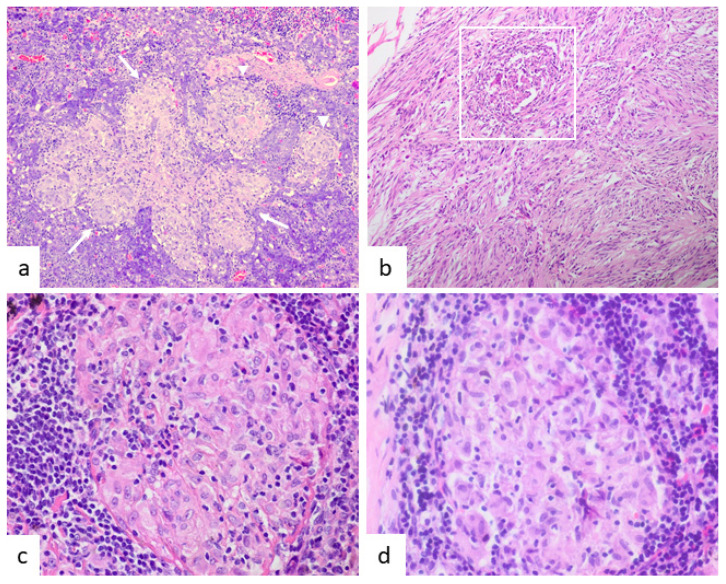
(**a**) Intratumoral granulomas were abundant in the case of acinic cell carcinoma of the parotid gland (case 1). A confluent granuloma (marked by three arrows) and two single granulomas (head arrows) are evident (HE, ×200). (**b**) In contrast, GIST showed a single intratumoral granuloma (white square) (HE, ×200). (HE, ×600). (**c**,**d**) Mediastinal lymph node from a patient with rectal adenocarcinoma (case 9) (**c**) and from a patient with sarcoidosis and no neoplasm (**d**). In both cases, morphology is similar, showing non-necrotizing, epithelioid granulomas with admixed lymphocytes (HE, ×400).

Concerning relevant analytical parameters, the C-reactive protein and erythrocyte sedimentation rate was increased in seven patients. Five had hypercalcemia and three had elevated levels of angiotensin-converting enzyme. The latter was not tested in all patients. The time interval between the diagnosis of sarcoidosis and the preceding neoplasia varied from 6 to 107 months with a mean period of 34 months. In nine patients, chest-imaging studies (four X-rays and five CT scans) were performed at the moment of neoplasm excision and showed no evidence of mediastinal lymphadenopathies or pulmonary involvement. The two patients with cutaneous tumors (neurofibroma and intraepidermal carcinoma) had no thoracic image study as excision was conducted using local anesthesia. At the time of the tumor excision, none of the 12 patients had symptomatology related to thoracic SS. In case 12, lymphoma with TAGs involved the mediastinum so there was no possibility of a previously normal imaging study. Nine of the twelve tumors were malignant neoplasms and three were benign (Figure 2, Figure 3, Figure 4 and Figure 5). It is remarkable that three of them were parotid gland tumors (Figure 2 and Figure 3a,b,e,f). 

In one case, TAGs were present in the primary tumor (rectal adenocarcinoma) and liver metastases (Figure 4c). Regarding the abundance of TAGs, in four cases they were a rare finding (Figure 5b); in six, moderate; and in two, abundant (Figure 5a). In only one case did intratumoral and peritumoral TAGs coexist. In five cases, the granulomas were exclusively intratumoral (Figure 2b, Figure 3e,f, Figure 4d and Figure 5a,b) and in the remaining six, only peritumoral (Figure 3a,d and Figure 4a–c). The squamous cell carcinoma of the tongue was well-differentiated and keratinizing, but the granulomas we considered were peritumoral, located distally from the main tumor, and showed no foreign body morphology or relationship with keratin (Figure 4a). A diagnosis of the mediastinal or pulmonary non-necrotizing granulomatous involvement was achieved by biopsy in five cases and by EBUS-TBNA cytology in the remaining seven. All of the histologic granulomas, tumor-associated, mediastinal, and pulmonary, were non-necrotizing and showed variable amounts of multinucleated giant cells, some of which contained asteroid and Schaumann bodies. Granulomas were accompanied by lymphocytes and occasionally eosinophils, but not neutrophils (Figure 5c,d). In all the cases, histochemical staining with Ziehl–Neelsen stain gave negative results. 

In the 65 controls, we found a granulomatous reaction in four cases. Two cases were present in squamous cell carcinomas of the tongue and were of the foreign body type. One was clearly related to the suture material of a previous biopsy, the second one was intratumoral, and had a close relationship with the keratin produced by the tumoral cells. A third case corresponded to liver metastasis of a mucinous intestinal adenocarcinoma, and the granulomatous reaction was of the foreign body type and related to mucoid material. Only one case of intestinal liver metastases showed focal peritumoral TAGs similar to the ones observed in case 7. We consider it a true SLR not associated with SS. In summary, in the cases group 12 patients (41.4%) showed “any granuloma” with no presence of the foreign body type granulomas. In the control group, four (6.2%) showed “any granuloma”, three of which were the foreign body type, while only one was an epithelioid classic granuloma. 

## 4. Discussion

In this study, some patients with TAGs were shown to later develop SS. To our knowledge, this association has not been previously reported. The study also shows that the relationship between SS and neoplasms extends to benign ones. Some studies describe sarcoid-like mediastinal lymphadenopathy or cardiac sarcoidosis in association with previous or concurrent cancer [12,13,14,15,16,17]. However, such studies do not include a histologic evaluation of previously diagnosed tumors in search of TAGs. We observed TAGs in a series of patients who months or years later developed SS. The number of TAGs in this study (12/29, 41,4%) is clearly higher than the general SLR ratio (between 4.4–13.8%) [10]. In our opinion, this is not a coincidental fact, and two possible explanations may justify this association. First, it may represent an initial phase of immune triggering against tumoral antigens that later expand in intensity and extension. Neoplasms accumulate genetic alterations, some of which produce mutated peptides that elicit T-cell responses [18]. These immunogenic mutated peptides, known as neoantigens, are foreign in nature and have tumor specificity. They are being used as promising targets to develop personalized clinical interventions [19]. Second, it can reflect the hyperreactive immune status of these patients to a variety of antigens, and that the development of the subsequent SS is not necessarily due to tumoral antigens. Whatever the cause, the fact is that tumors in patients with SS have an increased number of non-necrotizing granulomas and these can precede their diagnosis.

Cases of TAGs in patients with a previous diagnosis of SS have been reported [20,21,22], but we have found no descriptions of the inverse phenomenon: TAGs preceding the diagnosis of sarcoidosis. This last observation is interesting for several reasons. First, it reinforces the relationship between sarcoidosis and neoplasia, not only for malignant tumors but also benign ones. The medical literature regarding sarcoidosis and neoplasms is restricted to malignant tumors. If, as hypothesized, tumoral antigens are responsible for triggering the immune system of genetically predisposed individuals, it makes no difference if those antigens belong to a malignant or a benign tumor. In this sense, the lack of references is probably due to the relatively low clinical interest raised by benign tumors. We observed TAGs in patients with cutaneous neurofibroma, parotid oncocytoma and sebaceous lymphadenoma who later developed SS. Further studies will help us establish whether benign neoplasms are also associated with sarcoidosis. A second interesting point concerns the pathogenic mechanism underlying the association of cancer with subsequent sarcoidosis. In addition to tumoral antigens or neoantigens, it has been hypothesized that chemotherapy can predispose sarcoidosis development [23,24]. The most common drugs associated with granulomatous reactions are antiretroviral therapy, interferons, tumor necrosis factor-alpha antagonists, and immune checkpoint inhibitors [23,24]. However, in several studies, including ours, a relatively large percentage of patients received none of those drugs or chemotherapy. Rituximab is among the list of drugs that can induce a granulomatous reaction [23] and was given to three of our patients. In these patients, Rituximab could be related to thoracic sarcoidosis but not to TAGs, as these were present before treatment. From this, we could conclude that tumoral antigens could play an important role in the initiation of the immune activation even if there is a relatively large interval between the appearance of the tumor and the development of SS. In our study, the list of tumors with TAGs preceding sarcoidosis is highly variable, so no specific antigens can be identified. We observed them in benign and malignant epithelial and mesenchymal tumors, as well as lymphomas. Another interesting feature is the distribution of granulomas. In six cases, they were exclusively peritumoral, located in the normal tissue surrounding the tumor. This peritumoral location was observed in TAGs related or not to SS [17,22]. We therefore recommend performing a close examination of peritumoral tissue when trying to detect the presence of TAGs.

As the title of the study reflects, we must emphasize that in our series, TAGs preceded the “diagnosis” of SS as we cannot rule out the possibility of minimal mediastinal involvement at the time of tumor diagnosis. In this sense, the impossibility of having pathological proof of non-mediastinal granulomatous involvement at the time of the tumor diagnosis may be considered an intrinsic limitation of the study. Although thoracic X-rays may have a low sensitivity for the recognition of initial phases of SS, the five CT studies performed were normal and the patients had no related symptomatology. However, even if there was minimal subclinical involvement of mediastinal nodes at the time of tumor diagnosis, the main message of the study remains the same: patients with SS and previous tumors have a high incidence of TAGs. Other limitations are that this was a retrospective study, and our findings must be confirmed in prospective ones. Another limitation concerns the 18 patients from whom we had no pathologic sample available for review and the four we had to exclude as the simultaneous presentation of SS and neoplasia could not be ruled out. Although the pathologic analysis was not blinded, we consider that the inclusion of 65 control samples diminishes the possibility of granuloma detection bias.

A relevant observation of this study concerns mentioning the presence of TAGs in the final diagnosis of the pathologic report. Pathologists often describe them, but mostly they are not specifically mentioned in the final diagnosis. This is important because clinicians focus on the final pathologic diagnosis and many descriptions are not read. Adding “tumor-associated non-necrotizing granulomas are present” to the main diagnosis text may help the clinician consider SS if the patient subsequently develops mediastinal lymphadenopathies or pulmonary lesions. In conclusion, we observed TAGs in patients who months or years later develop SS. In our series, the ratio of TAGs in patients with SS was significantly greater than in the normal population, which reinforces a pathogenic relationship between SS and neoplasia. Since the introduction of EBUS-TBNA in clinical practice, the number of patients with a definite diagnosis of SS has increased. To prove our findings, we suggest reviewing the clinical history of patients diagnosed with SS in search of previous tumors. When present, such histologic slides should be reviewed in an attempt to identify TAGs.

## Figures and Tables

**Table 1:** Main clinicopathological features of patients with systemic sarcoidosis and preceding TAGs.

No	Age ^1^/Gender	Preexisting Neoplasm with Granulomas	Sarcoidosis Location and Clinical Stage	Time Elapse (months)	Granulomas Quantity	Granulomas Distribution	Follow up (months)	Extrathoracic Manifestations
1	55/M	Parotid gland acinic cell carcinoma	Mediastinal and lung, II	15	Abundant	Intratumoral	Alive (26), asymptomatic	-
2	59/F	Parotid gland oncocytoma	Mediastinal, I	30	Moderate	Peritumoral	Alive (51), asymptomatic	-
3	60/M	Bone marrow with lymphoma	Mediastinal, I	92	Abundant	Peritumoral	Alive (126), asymptomatic	-
4	72/M	Mediastinal lymphoma	Mediastinal and lung, II	40	Rare	Intratumoral	Died of neoplastic disease (54)	-
5	81/M	Parotid gland sebaceous lymphadenoma	Mediastinal, I	28	Moderate	Intratumoral	Alive, followed at another center	Uveitis
6	37/M	Squamous cell carcinoma of tongue	Mediastinal and lung, II	12	Rare	Peritumoral	Alive (122), asymptomatic	-
7	77/F	Rectal adenocarcinoma and liver metastases	Mediastinal and lung, II	7	Moderate	Peritumoral	Alive (158), asymptomatic	-
8	55/F	Cutaneous neurofibroma	Mediastinal and lung, II	22	Rare	Peritumoral	Alive (53), asymptomatic	Erythema nodosum
9	53/F	Rectal adenocarcinoma	Mediastinal, I	28	Moderate	Intratumoral and peritumoral	Alive (53), asymptomatic	-
10	81/M	Small bowel GIST	Mediastinal, I	16	Focal	Intratumoral	Alive (80), asymptomatic	-
11	70/F	Cutaneous intraepidermal carcinoma	Mediastinal, I	6	Moderate	Peritumoral	Alive (33), asymptomatic	-
12	87/F	Diffuse large B cell lymphoma	Mediastinal, I	107	Moderate	Intratumoral	Died of neoplastic disease (111)	-

Abbreviations: TAGs—tumor-associated granulomas, M—male, F—female, GIST—gastrointestinal stromal tumor, SS—systemic sarcoidosis. ^1^ Age at the time of sarcoidosis diagnosis.

## Data Availability

Data sharing not applicable. No new data were created or analyzed in this study.
